# Influence of Chain-Extension Reaction on Stereocomplexation, Mechanical Properties and Heat Resistance of Compressed Stereocomplex-Polylactide Bioplastic Films

**DOI:** 10.3390/polym10111218

**Published:** 2018-11-02

**Authors:** Yodthong Baimark, Sumet Kittipoom

**Affiliations:** 1 Biodegradable Polymers Research Unit, Department of Chemistry and Center of Excellence for Innovation in Chemistry, Faculty of Science, Mahasarakham University, Mahasarakham 44150, Thailand; 2 Faculty of Science, Mahidol University, 272 Rama VI Road, Ratchathewi, Bangkok 10400, Thailand; sumet.kii@mahidol.ac.th

**Keywords:** polylactide, stereocomplexation, chain extender, mechanical properties, heat resistance

## Abstract

Stereocomplex polylactide (scPLA) films were prepared by melt blending of poly(l-lactide) (PLLA) and poly(d-lactide) (PDLA) with and without an epoxy-based chain extender before compression molding. The obtained scPLA films were characterized through differential scanning calorimetry, X-ray diffractometry (XRD), tensile testing and dimensional stability to heat. XRD patterns revealed that all the scPLA films had only stereocomplex crystallites. The obtained results showed that the chain-extension reaction improved mechanical properties of the scPLA films, however, it suppressed stereocomplexation and heat resistance.

## 1. Introduction

Poly(l-lactic acid) or poly(l-lactide) (PLLA) is one of the most well-known biodegradable bioplastics, because of its low toxicity, bio-renewability, biocompatibility and good processability [1,2,3,4]; however, it has limited application because of its poor heat resistance [5,6]. Stereocomplex polylactides (scPLA) prepared by blending between PLLA and poly(d-lactide) (PDLA) have been widely investigated for use as high-performance bioplastics because the scPLA showed better mechanical properties, heat resistance and hydrolysis resistance compared to PLLA [7,8]. Highly heat-resistant scPLAs are required for specific applications, such as heat-treatment food packaging, hot fill packaging and microwave applications.

The scPLA have faster crystallization and higher melting points than PLLA due to the stereocomplex crystallites having stronger intermolecular forces than the homo-crystallites [9,10,11]. Stereocomplexation enhances the crystallization process of the scPLA, which improves its mechanical properties; for PLLA and PDLA, amorphous regions link among stereocomplex crystallites [12,13,14]. The heat resistance of scPLA is higher than in PLLA, because of faster crystallization and higher melting temperature of stereocomplex crystallites.

These scPLA films have been widely used in research, and are prepared by solution blending [7,8,11,15,16,17]. However, fabrication of scPLA films by melt processing is interesting, because of its possible use in industrial-scale applications. Specimens of scPLA have been prepared by injection [18] and extrusion [19,20]. For these purposes, PLLA and PDLA were melt blended within a screw barrel. To the best of our knowledge, scPLA films prepared by melt blending before compression molding has been scarcely published [21]. Therefore, stereocomplexation, mechanical properties and heat resistance of compressed scPLA films needs to be better understood so as to develop practical applications. Thermal chain-scission of PLLA and PDLA during melt blending and compression molding may reduce mechanical properties of the compressed films. Epoxy-based chain extenders have been used to maintain the molecular weight of the PLLA during the melt process through formation of long-chain branching structures [22,23,24]. However, chain-extension reaction of compressed scPLA films with various PLLA/PDLA ratios has not been reported so far.

Thus, this research work describes the influences of PLLA/PDLA ratio and chain-extension reaction on stereocomplexation, mechanical properties and heat resistance of compressed scPLA films.

## 2. Materials and Methods

### 2.1. Materials

l-Lactic acid (optical purity > 95%, Purac, Rayong, Thailand) and d-lactic acid (optical purity > 99%, Haihang Industry Co., Ltd., Jinan, China) were used as monomer precursors for synthesizing l-lactide (LLA) and d-lactide (DLA) monomers, respectively, by polycondensation, followed by thermal depolymerisation. These monomers were purified by re-crystallization four times from ethyl acetate before drying in a vacuum oven at 50 °C for 24 h. 1-Dodecanol (99%, Sigma-Aldrich, Buchs SG, Switzerland) was purified by distillation under reduced pressure before use. Stannous octoate (Sn(Oct)_2_, 95%, Sigma-Aldrich, Buchs SG, Switzerland) was used without further purification. Epoxy-based chain extender, Joncryl^®^ ADR 4368, in flake form was supplied by BASF, Bangkok, Thailand. All reagents used were analytical grade.

### 2.2. Synthesis and Characterization of PLLA and PDLA

High molecular-weight PLLA and PDLA were synthesized by ring-opening polymerization in bulk from the LLA and DLA, respectively, at 165 °C for 2.5 h under a nitrogen atmosphere using Sn(Oct)_2_ (0.01 mol %) and with 1-dodecanol (0.14 mol %) as the initiating system. The obtained PLLA and PDLA were granulated before drying in a vacuum oven at 110 °C for 3 h to remove the un-reacted monomers. The %conversions of both PLLA and PDLA determined from methine proton peaks of polylactide (5.1–5.3 ppm) and un-reacted lactide (4.9–5.1 ppm) of ^1^H-NMR spectra (Bruker Anvance 400 ^1^H-NMR spectrometer, Bruker, Billerica, MA, USA) were 99%. PLLA and PDLA were characterizaed by Waters e2695 separations module gel permeation chromatograph (GPC, Waters Corporation, Midford, MA, USA), ADP220 polarimeter (Bellingham and Stanley, Kent, UK) and Pyris Diamond differential scanning calorimeter (DSC, Perkin Elmer, Waltham, MA, USA). The results of PLA characteristics are reported in Table 1.

### 2.3. Preparation of scPLA and Their Compressed Films

PLLA, PDLA and Joncryl^®^ were dried in a vacuum oven at 50 °C overnight before melt blending using a HAAKE Polylab OS Rheomix batch mixer system (Thermo Fisher Scientific, Waltham, MA, USA) at 200 °C for 4 min with a rotor speed of 100 rpm. Effects of PLLA/PDLA blend ratios (100/0, 90/10, 80/20, 70/30 and 60/40 (*w*/*w*)) with (4.0 phr) and without Joncryl^®^ on properties of scPLAs were investigated. The obtained scPLAs were granulated before drying in a vacuum oven at 50 °C overnight before compression molding.

The compressed scPLA films were prepared using an Auto CH Carver laboratory press at 240 °C without any compression force for 1.0 min and with a 5 ton compression force for 1.0 min before quickly cooling to room temperature. The film thicknesses were in range 0.2–0.3 mm. The obtained films were kept at room temperature for 24 h before characterization.

### 2.4. Characterization of Compressed scPLA Films

The thermal transitions of compressed scPLA films were determined using a Pyris Diamond DSC (Perkin Elmer, Waltham, MA, USA) under a nitrogen atmosphere. For DSC, the thermal history of the samples was removed by melting at 250 °C for 3 min. Then, the sample was quenched to 0 °C before heating from 0 to 250 °C at a rate of 10 °C/min.

The crystalline structures of compressed scPLA films were investigated using a D8 Advance wide-angle X-ray diffractometer (XRD, Bruker, Billerica, MA, USA) at 25 °C with CuKα radiation at 40 kV and 40 mA. For XRD, a scan speed of 3°/min was chosen to determine the crystalline structures. The degrees of crystallinity from XRD (*X*_c,XRD_) of the scPLA films for homo-crystallites (hc-*X*_c,XRD_) and stereocomplex crystallites (sc-*X*_c,XRD_) were calculated by using Equations (1) and (2), respectively [20]:

hc-*X*_c,XRD_ (%) = S_hc_/(S_hc_ + S_sc_ + S_a_) × 100 (1)

sc-*X*_c,XRD_ (%) = S_sc_/(S_hc_ + S_sc_ + S_a_) × 100 (2)

where S_hc_, S_sc_ and S_a_ are the diffraction peak areas of homo-crystallites, stereocomplex crystallites and amorphous regions, respectively.

The tensile properties of compressed scPLA films were measured using a LRX + universal mechanical tester (Lloyd Instruments, West Sussex, UK) at 25 °C and 65% relative humidity. The films (100 × 10 mm) were tested with a gauge length of 50 mm and a crosshead speed of 50 mm/min. The tensile properties were averaged from at least five experiments for each sample.

The dimensional stability to heat of compressed scPLA films was tested at 80 °C for 30 s under a 200 g load. Initial length of film samples was 20 mm. The dimensional stability was calculated by Equation (3):

Dimensional stability (%) = [initial length (mm)/final length (mm)] × 100(3)

## 3. Results and Discussion

Compressed scPLA films with and without chain extension were prepared by melt blending followed with compression molding. The scPLA was chain-extended with Joncryl^®^ at the melt-blending step. The relationship of stereocomplexation, mechanical properties and dimensional stability to heat of the compressed scPLA films were studied using various analytical techniques.

### 3.1. Thermal Transition Properties

**Figure 1:**
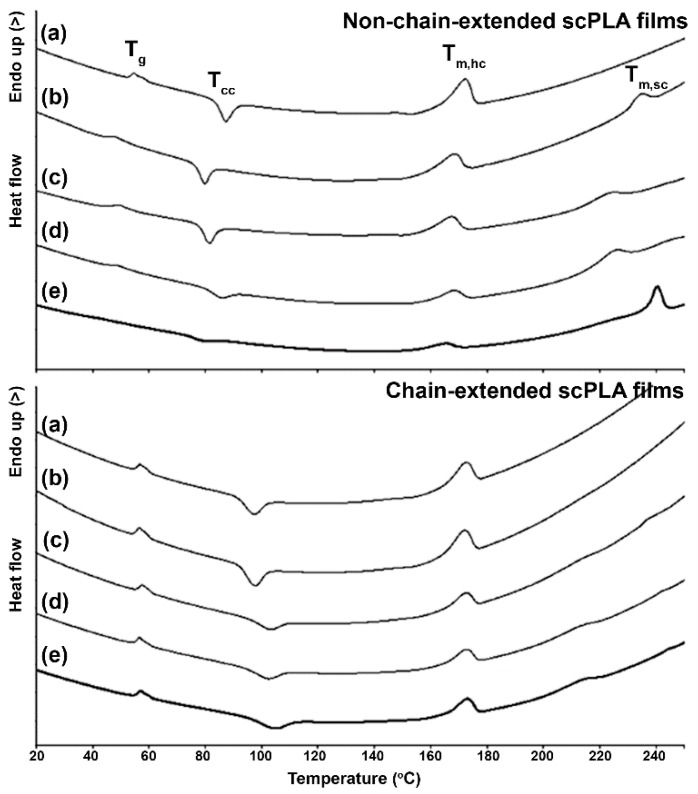
Heating DSC thermograms of (**above**) non-chain-extended and (**below**) chain-extended scPLA films with PLLA/PDLA ratios of (a) 100/0, (b) 90/10, (c) 80/20, (d) 70/30 and (e) 60/40 (*w*/*w*).

The thermal transitions of compressed scPLA films were determined from heating DSC thermograms as shown in Figure 1. The DSC results are summarized in Table 2. The *T*_g_ and *T*_cc_ of the chain-extended scPLA film series were higher than those of the non-chain-extended scPLA film series. This could be explained by long-chain branching structures of the chain-extended scPLA inhibiting chain mobility for glassy-to-rubbery transition and crystallization.

It is interesting that the Δ*H*_cc_ steadily decreased as the PDLA ratio increased for both the non-chain-extended and chain-extended scPLA film series suggesting that PDLA blending enhanced crystallization of the compressed scPLA films during film cooling. This is due to the crystallization of stereocomplex crystallites being faster than that of the homo-crystallites [7,11].

The *T*_m,hc_ and *T*_m,sc_ of compressed scPLA films were in the ranges 165–173 °C and 213–241 °C, respectively. The non-chain-extended 60/40 PLLA/PDLA exhibited the highest *T*_m,sc_. This may be due to the 40 wt % PDLA inducing the largest stereocomplex-crystallites. The Δ*H*_m,hc_ steadily decreased and Δ*H*_m,sc_ significantly increased when the PDLA ratios were increased indicating more stereocomplexation of scPLA film matrix. However, the chain-extended scPLA films had lower Δ*H*_m,sc_ than the non-chain-extended scPLA films for the same PDLA ratio. This suggests that the chain-extension reaction suppressed stereocomplexation of the PLA matrix. The degrees of crystallinity of both the homo- and stereocomplex crystallites of the compressed scPLA films could not be determined from DSC results because of the *T*_cc_ peaks of homo- and stereocomplex crystallizations overlapped each other [25].

### 3.2. Crystalline Structures

**Figure 2:**
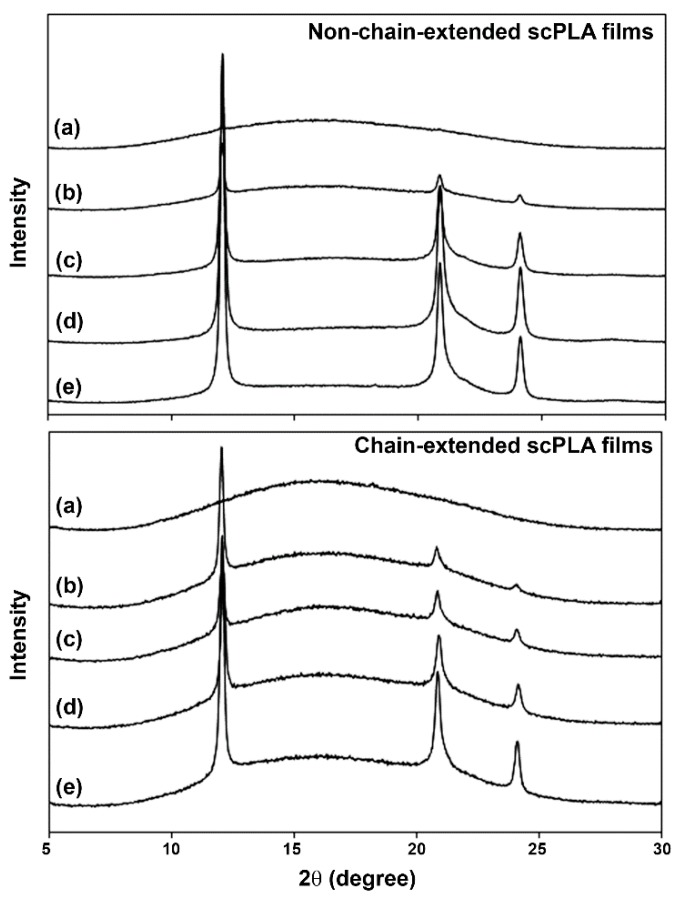
X-ray diffractometry (XRD) patterns of (**above**) non-chain-extended and (**below**) chain-extended scPLA films with PLLA/PDLA ratios of (a) 100/0, (b) 90/10, (c) 80/20, (d) 70/30 and (e) 60/40 (*w*/*w*).

The XRD patterns enabled determination of the crystalline structures of compressed scPLA films as shown in Figure 2. The XRD patterns of both the pure PLLA films with and without chain extension in Figure 2a exhibited a broad underlying ‘hump’ that was attributed to the XRD patterns for complete amorphous films. The XRD peaks w ere ascribed to crystalline fractions. For scPLA, the XRD peaks of 2*θ* = 15°, 17° and 19° were attributed to homo-crystallites while the XRD peaks of 2*θ* = 12°, 21° and 24° corresponded to stereocomplex crystallites [26,27]. It is interesting that all the compressed scPLA films with and without chain extension in Figure 2b–e showed only XRD peaks of stereocomplex crystallites. This may be explained by the compression force under high temperature (*T* > *T*_m,hc_) induced stereocomplex formation of the film matrix. It has been reported that external force can enhance sterecomplexation [15,17,28].

**Figure 3:**
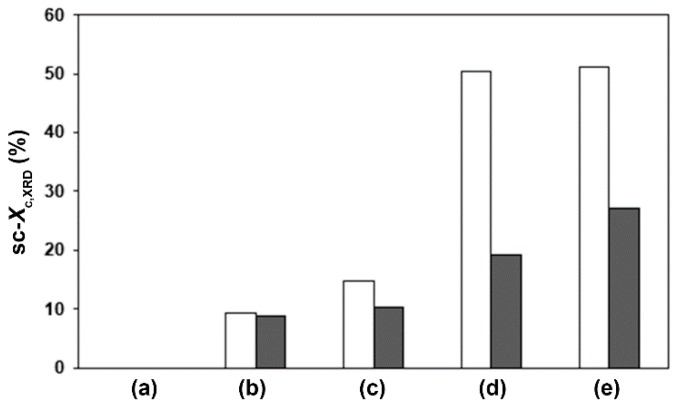
Stereocomplex crystallinity (sc-*X*_c,XRD_) values of (☐) non-chain-extended and (■) chain-extended scPLA films with PLLA/PDLA ratios of (a) 100/0, (b) 90/10, (c) 80/20, (d) 70/30 and (e) 60/40 (*w*/*w*).

It can be clearly seen that the intensities of the XRD peaks increased with the PDLA ratios, suggesting an increase of the stereocomplexation of compressed scPLA films. The value for sc-*X*_c,XRD_ can be calculated from the relative areas of the crystalline peaks and the amorphous hump with Equation (2). The values of sc-*X*_c,XRD_ are compared in Figure 3. The compressed PLLA films with and without chain-extension were completely amorphous as shown in Figure 3a (sc-*X*_c,XRD_ = 0%). The sc-*X*_c,XRD_ of both the compressed scPLA film series with and without chain-extension in Figure 3b–e increased with the PDLA ratios. The chain-extended scPLA films showed lower sc-*X*_c,XRD_ than the non-chain-extended scPLA films for the same PDLA ratio. This can be explained by long-chain branching structures of the chain-extended scPLA films that inhibited stereocomplexation according to the DSC results, as described above [29]. It should be noted that the *T*_m,hc_ peaks were detected with the DSC method though not with the XRD method. This may be explained by the homo-crystallization of PLA, which could have occurred during the DSC heating scan without compression forces. In addition, PLLA and PDLA homo-crystallization of scPLA films could have not occurred during compression molding at high temperature (*T* > *T*_m,hc_) [20].

### 3.3. Mechanical Properties

**Figure 4:**
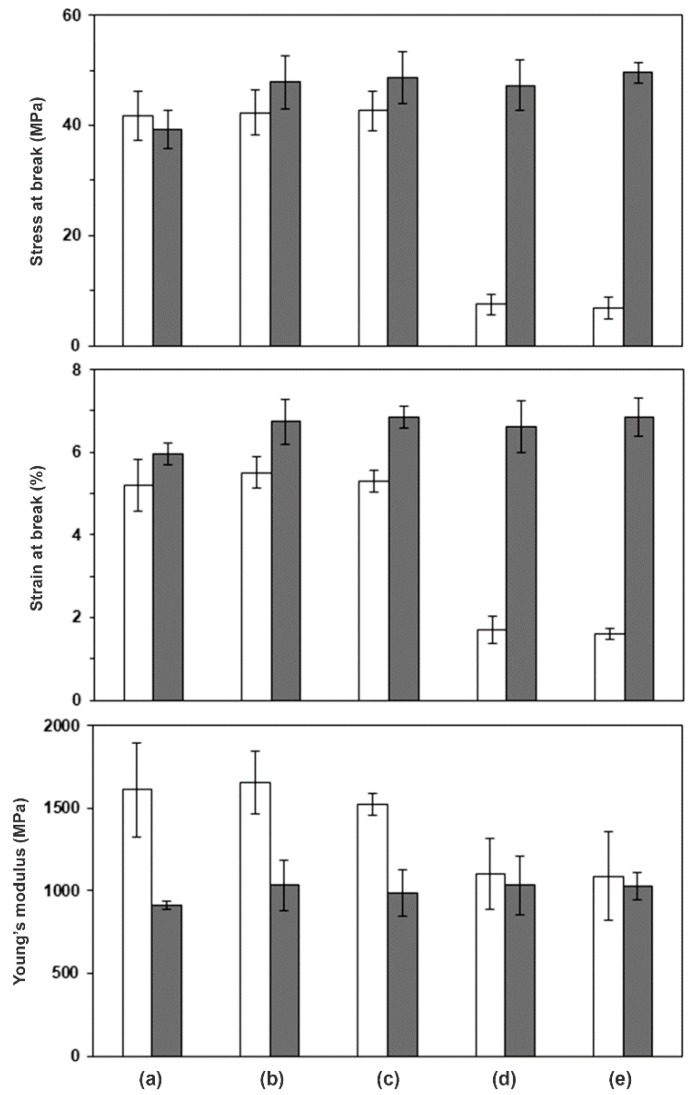
Tensile properties of (☐) non-chain-extended and (■) chain-extended scPLA films with PLLA/PDLA ratios of (a) 100/0, (b) 90/10, (c) 80/20, (d) 70/30 and (e) 60/40 (*w*/*w*).

Figure 4 shows the averaged values of tensile properties of the compressed scPLA films, including stress at break, strain at break and Young’s modulus. The stress and strain at break of non-chain-extended films with 0–20 wt % PDLA were in the ranges 41.7–42.3 MPa and 5.2–5.5%, respectively. It was found that the stress and strain at break of the non-chain-extended 80/20 PLLA/PDLA film in Figure 4c dramatically dropped from 42.7 to 7.5 MPa and 5.3 to 1.7%, respectively, when the PDLA ratio was increased up to 30 wt % (see Figure 4d). The results indicate that the non-chain-extended 70/30 PLLA/PDLA film was more brittle. This could be explained by the fact that higher sc-*X*_c,XRD_ values of non-chain-extended scPLA films caused film brittleness (see Figure 3). The DSC and XRD results suggested that the stereocomplexation between linear PLLA and PDLA of non-chain-extended scPLA was greater than between long-chain branched PLLA and PDLA of chain-extended scPLA.

However, the chain-extended scPLA film series showed higher stress and strain at break than the non-chain-extended scPLA film series. The results suggested that the chain-extension reaction improved stress and strain at break of compressed scPLA films. From our XRD results, the chain-extension reaction suppressed the sc-*X*_c,XRD_ of scPLA films by maintaining the molecular weights and branching formation of both the PLLA and PDLA. The longer PLLA and PDLA chains in amorphous regions which linked between sterecomplex crystallites could improve tensile properties of the scPLA films [14].

In addition, the stress and strain at the break of the chain-extended scPLA films in Figure 4b–e were higher than both the non-chain-extended and chain-extended PLLA films in Figure 4a. The Young’s modulus of non-chain-extended scPLA films decreased as the PDLA ratios increased, whereas the chain-extended scPLA films showed similar Young’s modulus in the range 915–1034 MPa.

### 3.4. Heat Resistance

**Figure 5:**
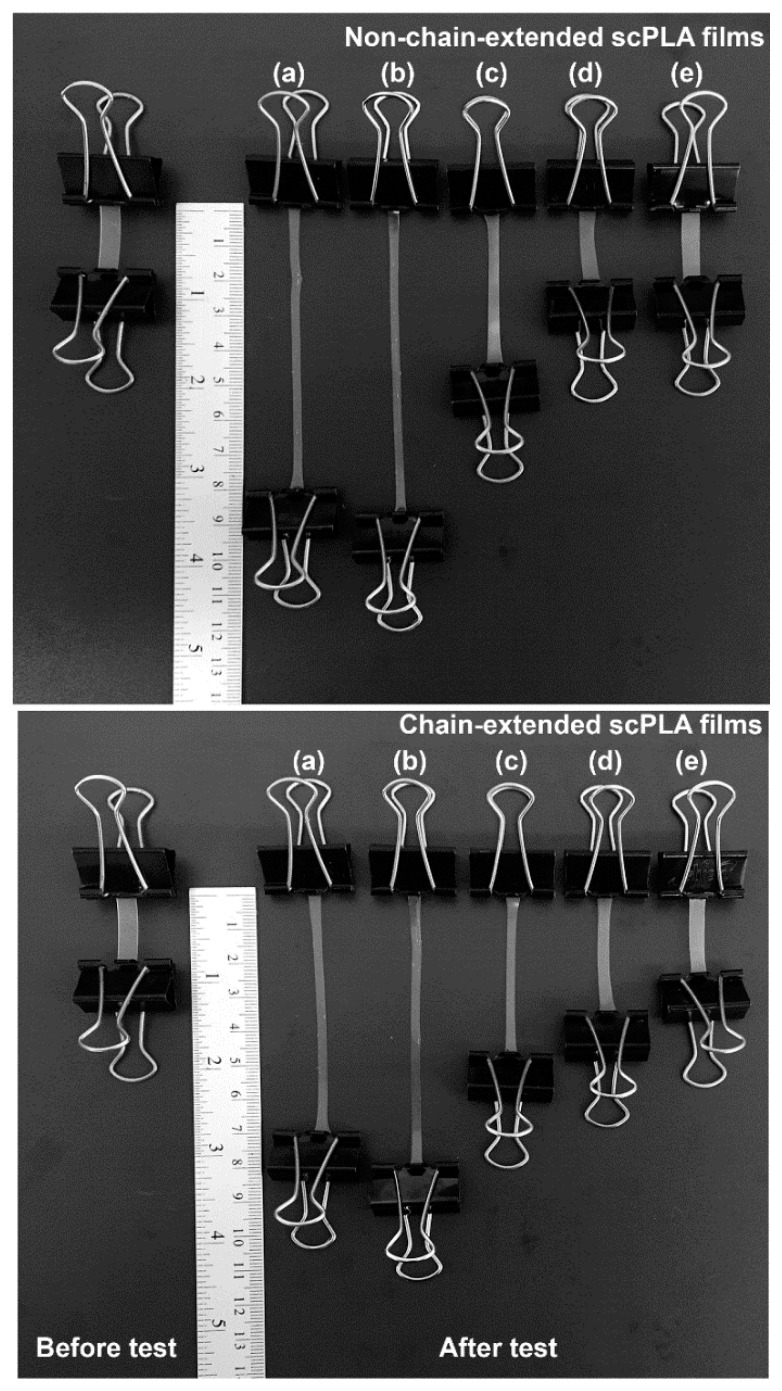
Dimensional stability to heat of (**above**) non-chain-extended and (**below**) chain-extended scPLA films with PLLA/PDLA ratios of (a) 100/0, (b) 90/10, (c) 80/20, (d) 70/30 and (e) 60/40 (*w*/*w*).

The dimensional stability to heat of compressed scPLA films was used to study the heat resistance of film samples. For this purpose, the films were kept at 80 °C under a 200 g load for 30 s High dimensional-stability of the film samples was attributed to its high heat-resistance. Figure 5 shows film samples before and after dimensional-stability testing. PLLA and 90/10 (*w*/*w*) scPLA films in Figure 5a,b showed similarly large final lengths for both the non-chain-extended and chain-extended films. The final film lengths significantly decreased as the PDLA ratio increased.

**Figure 6:**
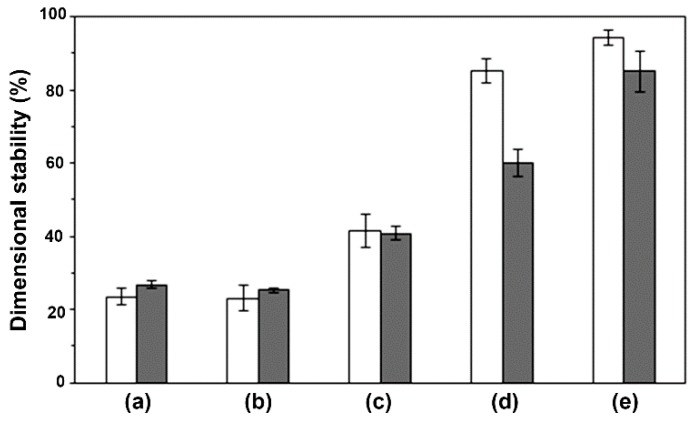
Dimensional stability to heat of (☐) non-chain-extended and (■) chain-extended scPLA films with PLLA/PDLA ratios of (a) 100/0, (b) 90/10, (c) 80/20, (d) 70/30 and (e) 60/40 (*w*/*w*).

The dimensional-stability values calculated from Equation (3) are clearly compared in Figure 6. The high heat-resistance polypropylene film prepared by the same compression molding had 100% dimensionally stability (not shown in Figure 6). The PLLA and 90/10 (*w*/*w*) scPLA films with and without chain-extension reaction in Figure 6a,b had similar dimensional stabilities in range 23.0–26.8%. They had poor heat-resistance because they had low sc-*X*_c,XRD_ (0–9.2%). The dimensional stabilities began increasing when the PDLA ratio was increased up to 20 wt % (Figure 6c). The film of non-chain-extended 60/40 (*w*/*w*) scPLA showed the highest dimensional stability (94.4%) because it had the largest sc-*X*_c,XRD_ (51.2%). The chain-extended scPLA films with PDLA ratios of 20–40 wt % exhibited lower dimensional-stabilities than the non-chain-extended films for the same PDLA ratio. This can be explained by the lower sc-*X*_c,XRD_ of chain-extended films inducing poor heat-resistance. Therefore, the heat resistance of scPLA strongly depended on its sc-*X*_c,XRD_.

## 4. Conclusions

In conclusion, the results reported here show that all the compressed scPLA films exhibited only stereocomplex crystallites as revealed by XRD. The sc-*X*_c,XRD_ of the compressed scPLA films significantly increased with the PDLA ratio. The chain-extension reaction suppressed stereocomplxation of the scPLA film matrices. However, the non-chain-extended scPLA films exhibited lower stress and strain at break than the chain-extended scPLA films. Therefore, the chain-extension reaction improved tensile properties of the compressed scPLA films. The dimensional stabilities to the heat of compressed scPLA films without chain-extension were as high as 85% and 94% for 30 and 40 wt % PDLA, respectively. The chain-extension reaction reduced dimensional stabilities to the heat of compressed scPLA films containing 30 and 40 wt % PDLA. The chain-extension reaction decreased both the stereocomplexation and heat resistance of the scPLA films. This work could provide guidance toward fabrication of compressed scPLA products with balanced stereocomplexation, mechanical properties and heat resistance and wider applicability for scPLA.

## Figures and Tables

**Table 1:** Characteristics of poly(l-lactide) (PLLA) and poly(d-lactide) (PDLA).

PLA	L Enantiomer Content ^a^ (%)	*M*_n_^b^ (g/mol)	DI ^b^	*T*_g_^c^ (°C)	*T*_m_^c^ (°C)
PLLA	96.4	85,000	2.1	54	173
PDLA	3.2	90,000	2.8	59	176

^a^ determined from polarimetry using CHCl_3_ as the solvent at 25 °C with a wavelength of 589 nm. ^b^ *M*_n_ (number-averaged molecular weight) and dispersity index (DI) measured by GPC using tetrahydrofuran as the eluent at 40 °C. ^c^ glass transition temperature (*T*_g_) and melting temperature (*T*_m_) measured by differential scanning calorimetry (DSC) (PLLA and PDLA samples were melted at 200 °C for 3 min and cooled to 0 °C before scan from 0 to 200 °C at 10 °C/min under N_2_ flow).

**Table 2:** Thermal transition properties of stereocomplex polylactide (scPLA) films.

PLLA/PDLA Ratio (*w*/*w*)	*T*_g_ (°C)	*T*_cc_^a^ (°C)	Δ*H*_cc_^a^ (J/g)	*T*_m,hc_^a^ (°C)	Δ*H*_m,hc_^a^ (J/g)	*T*_m,sc_^a^ (°C)	Δ*H*_m,sc_^a^ (J/g)
Non-Chain-Extended Films							
100/0	52	87	28.4	172	52.5	-	-
90/10	44	80	21.4	168	38.9	234	16.3
80/20	44	81	20.1	167	32.7	223	19.6
70/30	45	86	14.0	168	19.7	225	27.0
60/40	41	79	6.7	165	11.4	241	30.6
Chain-extended films							
100/0	54	97	25.9	172	32.9	-	-
90/10	54	98	24.2	172	33.9	-	-
80/20	55	103	16.4	172	21.4	213	2.7
70/30	54	102	16.2	173	21.2	213	6.2
60/40	54	105	16.3	173	19.8	214	9.2

^a^*T*_cc_ = cold crystallization temperature, Δ*H*_cc_ = enthalpy of cold crystallization, *T*_m,hc_ = melting temperature of homo-crystallites, Δ*H*_m,hc_ = enthalpy of homo-crystallite melting, *T*_m,sc_ = melting temperature of stereocomplex crystallites and Δ*H*_m,sc_ = enthalpy of stereocomplex-crystallite melting.
